# Storage Conditions and Adsorption Thermodynamic Properties for Purple Corn

**DOI:** 10.3390/foods11060828

**Published:** 2022-03-14

**Authors:** David Choque-Quispe, Betsy S. Ramos-Pacheco, Yudith Choque-Quispe, Rolando F. Aguilar-Salazar, Antonieta Mojo-Quisani, Miriam Calla-Florez, Aydeé M. Solano-Reynoso, Miluska M. Zamalloa-Puma, Ybar G. Palomino-Malpartida, Tarcila Alcarraz-Alfaro, Alan Zamalloa-Puma

**Affiliations:** 1 Department of Agroindustrial Engineering, Universidad Nacional José María Arguedas, Andahuaylas 03701, Peru; bsramos@unajma.edu.pe; 2 Department of Environmental Engineering, Universidad Nacional José María Arguedas, Andahuaylas 03701, Peru; ychoque@unajma.edu.pe; 3 Department of Basic Sciences, Universidad Nacional José María Arguedas, Andahuaylas 03701, Peru; raguilar@unajma.edu.pe; 4 Department of Agroindustrial Engineering, Universidad Nacional de San Antonio Abad del Cusco, Cusco 08000, Peru; antonieta.mojo@unsaac.edu.pe (A.M.-Q.); miriam.calla@unsaac.edu.pe (M.C.-F.); 5 Department of Environmental Engineering, Universidad Tecnológica de los Andes, Andahuaylas 03701, Peru; ayma_21@hotmail.com; 6 Department of Physics, Universidad Nacional de San Antonio Abad del Cusco, Cusco 08000, Peru; miluska.zamalloa@unsaac.edu.pe (M.M.Z.-P.); alan.zamalloa@unsaac.edu.pe (A.Z.-P.); 7 Department of Chemical Engineering, Universidad Nacional de San Cristobal de Huamanga, Ayacucho 05000, Peru; ybar.palomino@unsch.edu.pe (Y.G.P.-M.); tarcila.alcarraz@unsch.edu.pe (T.A.-A.)

**Keywords:** purple corn, adsorption isotherm, isosteric heat, Gibbs free energy, differential entropy, activation energy, isokinetic theory

## Abstract

Adsorption isotherms provide insight into the thermodynamic properties governed by food storage conditions. Adsorption isotherms of purple corn of the Canteño variety were evaluated at 18, 25, and 30 °C, for the equilibrium relative humidity (ERH) range between 0.065 and 0.95. The equilibrium moisture (*X_e_*) was determined by the continuous weight-change method. Seven mathematical models of isotherms were modeled, using the coefficient of determination R^2^, mean absolute error (MAE), and estimated standard error (ESE) as the convergence criterion. Thermodynamic parameters such as isosteric heat (*q_st_*), Gibbs Free Energy (Δ*G*), differential entropy (Δ*S*), activation energy (*E_a_*), and compliance with the isokinetic law were evaluated. It was observed that the adsorption isotherms presented cross-linking around 75% ERH and 17% *X_e_*, suggesting adequate storage conditions at these values. The GAB and Halsey models reported better fit (R^2^ > 97%, MAE < 10%, ESE < 0.014 and random residual dispersion). The reduction of *X_e_* from 17 to 7%, increases *q_st_*, from 7.7022 to 0.0165 kJ/g, while Δ*G* decreases considerably with the increase in *X_e_*, presenting non-spontaneous endergonic behavior, and linear relationship with Δ*S*, evidencing compliance with the isokinetic theory, governed by *q_st_*. *E_a_* showed that more energy is required to remove water molecules from the upper layers bound to the monolayer, evaluated using *C_GAB_*. The models predicted the storage conditions, and the thermodynamic parameters show the structural stability of the purple corn grains of the Canteño variety during storage.

## 1. Introduction

Pigments in the grains of purple corn (*Zea mays* L.), in addition to being used as natural colorants, are attributed biological functions as antioxidants [1,2], and are found in mainly the pericarp, aleurone, endosperm, and embryo of corn [3,4,5,6]. These compounds present nutritional interest for their contribution to human health due to their beneficial properties [7,8]. Similarly, purple corn, due to its color, is used in food, cosmetic and pharmaceutical products [9,10,11], and is widely consumed in countries such as Peru, Bolivia, Ecuador, and Mexico, especially in porridge, desserts, and as a drink, due to its pleasant flavor and striking color [12,13,14].

However, the functional or antioxidant properties, which purple corn grains present, may be susceptible to changes, even losing their qualities due to storage conditions, such as inadequate temperature and relative humidity [4,15], and the uncontrolled combination of these can allow for the development of molds and yeasts [16,17,18,19], or at the other extreme allow weight loss, which would cause economic losses due to low humidity or the deterioration of the grain due to cracking and wear of the food surface [20,21].

The equilibrium water content (*X_e_*) in food is reached when the partial vapor pressure of the material equals the vapor pressure of the air that contains it, to the ratio of the vapor pressure of the food. The ambient air is called water activity, *a_w_* and is a determining factor during storage [22,23].

Numerous physicochemical, semi-empirical, and empirical mathematical models have been developed that help to study the adsorption behavior of water in foods [24,25,26], in equilibrium with the atmosphere that contains it, at different storage temperatures, called adsorption isotherms [27,28,29], describing the behavior of water at the level of a monolayer (BET isotherm), multilayer (GAB isotherm) [30,31,32], or simply by adjusting *a_w_* and equilibrium moisture data [33,34]. These models, such as BET and GAB, provide information on the thermodynamic behavior of the water bound to the active sites [35,36], on the surface of the food.

On the other hand, isotherms provide information on thermodynamic adsorption parameters, which are useful for the design of drying and storage equipment [37,38,39], thus, the isosteric heat of sorption is an indicator of the bond strength between free water and the surface of the food, and the higher this is, the greater the energy required during drying [40,41]. Another aspect to consider is the speed with which water molecules dissipate in the active sites of materials or foods, which is related to entropy [26,42]. This movement of water molecules facilitates the vaporization process, which can occur spontaneously, and can be measured using the Gibbs free energy [43,44].

There is currently a great interest in consuming foods with minimal processing, with high nutritional value, and that also provide health benefits [45], such as purple corn; however, these are susceptible to deterioration and loss of functionality during storage, which would generate economic losses in the producer and marketer, for this reason, the research aimed to study the storage conditions and thermodynamic properties of purple corn grains.

## 2. Materials and Methods

### 2.1. Samples

Grains of purple corn (*Zea mays* L.) of the Canteño variety, dried outdoors, were used, with an initial humidity of 11.03% dry basis (d.b.), produced in the fields of the José María Arguedas National University, Santa Rosa farm at 2804 m altitude, 13°39′05″ S and 73°26′31″ W, in the province of Andahuaylas, Peru.

### 2.2. Construction of Adsorption Isotherms

The construction of the adsorption isotherms was based on the static gravimetric method [46]. Nine glass jars of 200 mL with hermetic lid were conditioned, with a tripod incorporated as the support where three corn grains were placed. Previously, the flasks were loaded with saturated solutions of chemical substances with water activity values between 0.06 and 0.92 (Table 1).

The jars were placed in a Memmert model 100–800 stove at 18, 25, and 30 °C. Weighing of the corn grains was carried out every three days with precise analytical balance until the samples presented a constant weight, that is, they reached equilibrium with their atmosphere. Sodium azide at 0.25% was added to prevent microbiological growth and grain germination for water activities above 0.5.

### 2.3. Determination of Equilibrium Moisture

The equilibrium humidity was calculated by the difference between the mass of the sample that reaches equilibrium and the dry mass, according to equation:

(1) $$X_{e}=\frac{m_{\mathrm{eq}}-m_{s}}{m_{s}},$$

where, *X_e_* is the equilibrium moisture on a dry basis; *m*_eq_, is the mass of the sample at equilibrium, g; and *m_s_* is the mass of the dry sample, g.

### 2.4. Adjustment of Adsorption Isotherms

The experimental data were fitted to adsorption isotherm models (Table 2), by non-linear regression, applying the Quasi-Newton method, using Statistica 8.0 Software (Statsoft, Tulsa, OK, USA). The goodness of fit was evaluated using the fit coefficient R^2^, mean absolute error (MAE) (Equation (2)) and the estimated standard error (ESE) (Equation (3)), by considering good fit when MAE < 10% and ESE lower [47,48,49,50,51]. Likewise, the dispersion of the residuals of *X_e_* was taken as a convergence criterion, which evaluates the tendency of the systematic and random errors during the experimentation [51].

(9) $$\%\mathrm{MAE}=\frac{100}{N}*\sum_{i=1}^{n}\left|\frac{{\mathrm{Me}}_{i,\exp}-{\mathrm{Me}}_{i,\mathrm{pre}}}{{\mathrm{Me}}_{i,\exp}}\right|,$$

(10) $$\mathrm{ESE}=\sqrt{\frac{\sum_{i=1}^{N}{\left({\mathrm{Me}}_{i,\exp}-{\mathrm{Me}}_{i,\mathrm{pre}}\right)}^{2}}{N-n}},$$

where, M_ei,exp_ is the observed experimental equilibrium moisture content; M_ei,pre_ is the predetermined moisture content in the observations; *N* is the number of experimental observations, *n* is the number of constants in the model.

### 2.5. Thermodynamic Parameters

The isosteric heat of adsorption (*q_st_*) (or differential enthalpy) evaluates the difference between the total heat of sorption in the purple corn and the heat of vaporization of water at the system temperature [52], and can be estimated using the Clausius-Clapeyron equation (Equation (11)) [53].

The value of *q_st_* was obtained by plotting ln *a_w_* vs. 1/*T*, at their respective humidities, where *q_st_*/*R* is the slope.

(11) $${\frac{\partial \ln\left(a_{w}\right)}{\partial (1/T)}|}_{x}=-\frac{q_{st}}{R}$$

where, $a_{w}$ is the water activity; *T* is the absolute temperature (K); *q_st_* is the isosteric heat of sorption (kJ/kg); and *R* is the universal gas constant (8.314 kJ/kmol·K) for water (0.4619 kJ/kg·K).

On the other hand, the *q_st_* data *X_e_*, were fitted to the Tsami equation (Equation (12)) [53].

(12) $$q_{st}=q_{0}\exp(-\frac{X_{e}}{X_{0}})$$

where, *q_st_* is the isosteric heat of sorption when the moisture content is constant; *X_e_* is the equilibrium humidity (g water/g dry sample), *q*_0_ is the isosteric heat of adsorption (kJ/mol) of the first water molecule in the food and is defined as *X_e_* → 0 ⇒ *q_st_* → *q*_0_; and *X*_0_ is the characteristic moisture content for each product.

The differential entropy of sorption (Δ*S*) (kJ/kg·K) was calculated using the Gibbs–Helmholtz equation (Equation (13)) [54].

(13) $$\Delta S=\frac{q_{st}-\Delta G}{T}$$

where Δ*G* is the Gibbs free energy (kJ/kg), it is expressed using Equation (14).

(14) $$\Delta G=-RTln\left(a_{w}\right)$$

During the adsorption process, the variation of Gibbs free energy is related to the variation of isosteric heat and entropy, thus by replacing Equation (14) in (13), Equation (15) is obtained.

(15) $$-\ln\left(a_{w}\right)=\frac{q_{st}}{RT}-\frac{\Delta S}{R}$$

The linear form of Equation (15), allows us to obtain the intercept and calculate Δ*S*.

The enthalpy–entropy compensation theory suggests the existence of a linear relationship between enthalpy and entropy according to Equation (16) [36,55,56].

(16) $$q_{st}=T_{\beta }\Delta S+\Delta G_{\beta }$$

where, *T_β_* is the isokinetic temperature (K); Δ*G_β_* is the free energy (kJ/kg) at *T_β_*.

*T_β_* is an indicator in which it is assumed that all interactions within the purple corn grains occur with the same speed [57], while the term +Δ*G_β_* represents whether the adsorption process is spontaneous or not (−Δ*G_β_*).

The validity of the compensation theory was evaluated by comparing *T_β_* with the harmonic mean temperature (*T_hm_*) (Equation (17)) [56,58,59], and it is valid when *T_β_* ≠ *T_hm_*, likewise if *T_β_* > *T_hm_*, the process of sorption is governed by the isosteric heat of sorption (enthalpy of sorption), and if *T_β_* < *T_hm_* by the entropy [60,61].

(17) $$T_{hm}=\frac{n}{\sum_{i=1}^{n}1/T}$$

where, *n* is the number of used temperatures.

The effect of temperature on humidity was evaluated using the Arrhenius equation (Equation (18)), for the GAB isotherm parameters.

(18) $$\ln(D)=\ln(D_{0})-\frac{E_{a}}{RT}$$

where, *D* is a parameter of the GAB model, *D*_0_ is a pre-exponential factor, and *E_a_* is the activation energy (kJ/mol).

## 3. Results and Discussion

### 3.1. Adsorption Isotherms

**Figure 1:**
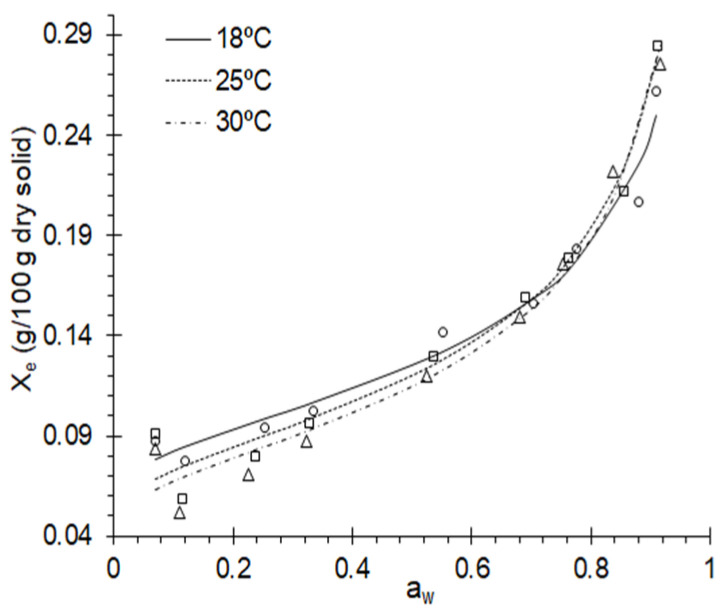
Adsorption isotherms adjusted with the Halsey model.

An equilibrium moisture, *X_e_*, of purple corn was reached after 15 days at 18 °C, and in 12 days at 25 °C and 30 °C. The behavior of *X_e_* at storage conditions is shown in Figure 1, and a crossover of the isotherms around *a_w_* 0.75 is observed, due to the composition of purple corn of a higher content of carbohydrates and sugars compared to other corn varieties, this behavior is characteristic of fruits with a high sugar content [16,20,22,23,25,31,62].

Likewise, the increase in temperature would promote the availability of active sites to adsorb water on the corn grain surface, due to the effects caused by capillarity and humidity interactions [23,62,63], this being a typical behavior of a type II isotherm [27,33,64,65].

### 3.2. Adjustment of Adsorption Isotherms

It was observed that, at 18 °C, the GAB and Halsey models reported R^2^ values of 0.967 and 0.974, MAE of 5.149% and 5.902%, and ESE 0.013 and 0.011, respectively; at 25 °C, R^2^ values of 0.973 and 0.976, MAE 8.795% and 8.628% and ESE 0.014 and 0.012 were found, while at 30 °C, R^2^ values were reported to be 0.984 and 0.975, MAE 8.508% and 10.412% and ESE as 0.011 and 0.013, respectively (Table 3). In the same way, both models presented random residual dispersion at the study temperatures, which indicates that the models better attenuate systematic and experimental errors due to repetitiveness, better representing the adsorption phenomenon [22,25,34,66,67].

On the other hand, the Oswin, Modified Henderson, Chun-Pfost, and Henderson models reported R^2^ values > 0.90. In fact, these models are used as predictors of *X_e_* behavior at different relative humidities, generally for cereals and fruits [20,30,35,44,65].

Regarding the *C_GAB_* values, these were greater than unity, which indicates that the adsorption in the monolayer is fast, that is, the humidity at the monolayer level is achieved quickly during the first days and, as a consequence, the purple corn is prone to rapid attack by molds and yeasts [26,27,68].

Furthermore, *C_GAB_* is high is because the surface of the purple corn grain is constituted by a large number of active centers, including polar groups of the −CO, −COO^−^ and −NH^3+^, which allow it to establish a greater number of hydrogen bridge bonds.

On the other hand, the parameter *k_GAB_*, which is related to the standard chemical potential between the molecules of the second layer and those of the pure liquid state, was observed to increase with temperature (Table 3), which suggests a decrease in humidity at low *a_w_* values.

While moisture at the monolayer *X_m_* level of the GAB model, was found to be around 7% d.b., which is a usual behavior for corn varieties [39,69], the fact that *X_m_* decreases with temperature indicates that at higher temperatures, the moisture loss is greater at the monolayer level for a defined relative humidity [67,69], due to the breaking of the intermolecular bonds of the hydrogen bridge type between the surface of the corn grain and the water available at the *X_e_* level. This suggests that temperature is a critical condition for the attack of molds and yeasts, which is a typical behavior of foods that follow type II isotherms [24,34,36,39,54,64].

### 3.3. Thermodynamic Parameters

**Figure 2:**
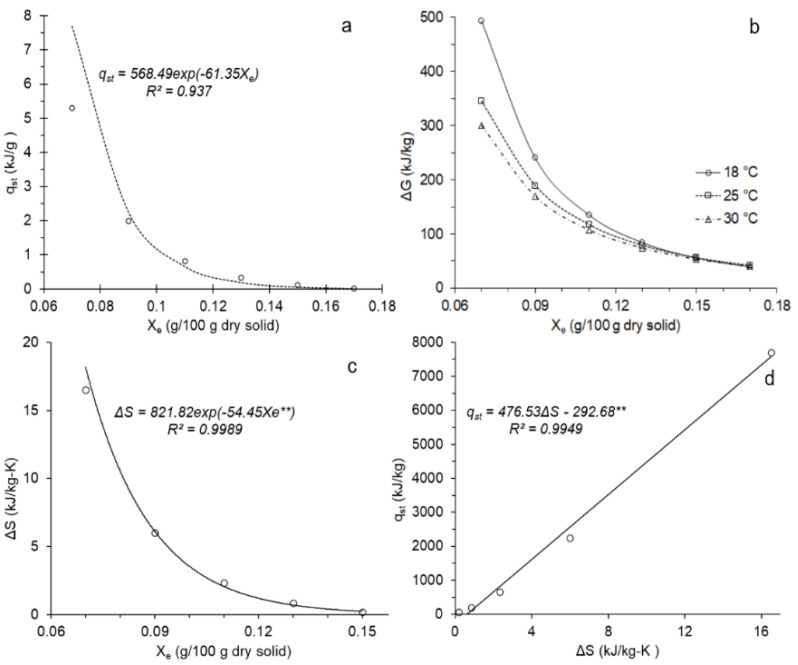
(**a**) Isosteric heat of sorption; (**b**) Gibbs free energy; (**c**) Differential entropy for purple corn (**d**) Relationship between differential entropy and isosteric heat of sorption (** evaluated at 5% significance).

The isosteric heat of sorption *q_st_* of purple corn was determined considering *a_w_* values calculated using the Halsey equation, for *X_e_* between 0.07 and 0.17. Figure 2a, presents the behavior of *q_st_* as a function of *X_e_*, and it is observed that as the equilibrium humidity increases, the value of *q_st_* decreases from 7.7022 to 0.0165 kJ/g, meaning this behavior is a result of an initially high humidity at the monolayer level, requiring more energy to break the polar and hydrogen bonds on the surface of the corn grain, and as *X_e_* increases, the active sites that adsorb water are no longer available, which is usual in foods with a high carbohydrate content [39,67,69,70,71].

The *q_st_* values found are higher than those reported for this cereal [39,69] due to the coloration of the purple corn grain, which is related to the presence of phenolic compounds and sugars [2,5,15], thereby giving it a greater number of functional groups, with the capacity to establish a greater number of bonds with water, for which it would require more energy to eliminate it from the monolayer.

The work required to make sorption active sites available was calculated using the Gibbs free energy, this being a thermodynamic indicator between the corn grain and the water [72]. Furthermore, it was observed that it decreases with the increase in temperature and humidity. At 7% humidity, Δ*G* was 493.82, 344.49, and 300.67 kJ/kg at 18, 25, and 30 °C, respectively, this significant change is observed at up to 15% humidity, for which similar values of Δ*G* are observed (Figure 2b). Furthermore, this behavior is characteristic of grains and cereals [35,39,41,42,73,74] because the available sites on the surface of the purple corn grain have been occupied, consistent with the crossing of the isotherms (Figure 1).

On the other hand, it was observed that Δ*G* > 0, suggesting an endergonic process, that is, a driving force, is required to initiate the binding of water molecules during adsorption, and that as *X_e_* increases, the availability to form bonds is lower, thereby requiring less energy, which is characteristic of non-spontaneous processes when they reach equilibrium, as evidenced by the adsorption systems of purple corn at different relative humidities [41,54,75].

The availability of active sites depends on how fast water molecules are mobilized on the surface of the purple corn grain, and this was calculated using differential entropy (Δ*S*) [24,42]. It was observed that Δ*S* decreases from 16.51 to 0.19 kJ/kg·K for the X_e_ interval between 7 to 15%, presenting a rapid drop up to *X_e_* 11% (Figure 2c), this would be due to the greater availability of the active sites, and from this point, the mobility of the molecules decreases, related to Δ*G* [19,41,76].

Similarly, a linear relationship (R^2^ > 0.99) was observed between *q_st_* and Δ*S* (Figure 2d), that is, there is a direct relationship between the energy needed to bind free water to the food surface, and the mobility of water molecules at the monolayer level, so the isokinetic theory, or enthalpy–entropy compensation, applies to this experimentation [36,45,55,56].

The isokinetic temperature *T_β_* was 476.53 K, while *T_hm_* 297.0 K, which suggests a sorption process governed by *q_st_* (*T_β_* > *T_hm_*) [60,61], which is usual behavior in seeds and grains [24,35,41,43], likewise, this comparison established that purple corn grains remain stable following structural modifications that could occur during water removal or drying in the range of the study temperatures [44,77].

The GAB isotherm parameters have a thermodynamic interpretation via the activation energy (*E_a_*), which represents the necessary energy of the phenomena occurring at the level of the water monolayer of the corn grain surface.

Thus, the energy for water to be adsorbed towards the surface of the corn grain, to form the monolayer (*X_m_*), and bind to the specific polar groups of corn, was 10.947 kJ/mol, for the interval from 18 to 30 °C (Table 4), on the other hand, the *C_GAB_* parameter is related to the difference in energy of the molecules adsorbed in the monolayer and the upper ones [27,44,56,68], whose value was 18.84 kJ/mol. Likewise, the parameter *k_GAB_*, which refers to the chemical potential, that is, the energy necessary to form the bond between the water molecules and the active sites [68], was 6.82 kJ/mol.

## 4. Conclusions

Adsorption isotherms presented crosslinking at around 75% RH and 17% *X_e_* at 18, 25, and 30 °C, suggesting adequate storage conditions at these values. The GAB and Halsey models reported a better fit and would allow for a description of the behavior of corn grain moisture at different equilibrium relative humidities. The reduction of *X_e_* between 17 and 7% occurs with an increase in the isosteric heat of adsorption, *q_st_*, from 7.7022 to 0.0165 kJ/g, while the Gibbs free energy decreases considerably with the increase in *X_e_* at the study temperatures, showing a non-spontaneous endergonic behavior, and presents a positive linear relationship with the adsorption differential entropy, evidencing the compliance of the isokinetic theory, governed by *q_st_*, which suggests the structural stability of corn grains during storage and drying. The activation energy showed that more energy is required to remove water molecules from the upper layers bound to the monolayer, evaluated using *C_GAB_*.

## Figures and Tables

**Table 1:** The water activity of substances for the construction of isotherms.

Substance	Equation	R^2^
Sodium hydroxide	$a_{w}=0.081-1.128\times 10^{-3}T+3.929\times 10^{-5}T^{2}-5.092\times 10^{-7}T^{3}$	0.998
Lithium chloride	Ln aw=(500.95T)−3.85	0.980
Potassium Acetate	Ln aw=(861.39T)−4.33	0.970
Magnesium chloride	$a_{w}=0.365-2.523\times 10^{-3}T+5.071\times 10^{-5}T^{2}-4.166\times 10^{-7}T^{3}$	0.963
Magnesium Nitrate	Ln aw=(356.60T)−1.82	0.990
Potassium iodide	Ln aw=(255.90T)−1.23	1.000
Sodium chloride	Ln aw=(228.92T)−1.04	0.960
Potassium chloride	Ln aw=(367.58T)−1.39	0.970
Barium chloride	$a_{w}=0.908-4.011\times 10^{-4}T+2.786\times 10^{-5}T^{2}-2.037\times 10^{-7}T^{3}$	0.997

$a_{w}$, is the water activity; *T* is the temperature (K). Source: Labuza et al. [46].

**Table 2:** Mathematical models of the adsorption isotherm.

Model		
Temperature dependent		
BET	$x_{e}=\frac{x_{m}c_{BET}a_{w}}{\left[\left(1-a_{w}\right)\left(1+\left(c_{BET}-1\right)a_{w}\right)\right]}$	(2)
GAB	$x_{e}=\frac{x_{m}c_{GAB}k_{GAB}a_{w}}{\left[\left(1-k_{GAB}a_{w}\right)\left(1-k_{GAB}a_{w}+c_{GAB}k_{GAB}a_{w}\right)\right]}$	(3)
Oswin	$x_{e}=A{\left[\frac{a_{w}}{1-a_{w}}\right]}^{B}$	(4)
Modified Henderson	$1-a_{w}=\exp(-kTx_{e}^{n^{\prime }})$	(5)
Chung y Pfost	$a_{w}=\exp(\frac{A}{RT}\exp(-Bx_{e}))$	(6)
Temperature independent		
Halsey	$a_{w}=\exp\left[\frac{-A}{x_{e}^{B}}\right]$	(7)
Henderson	$1-a_{w}=\exp(-kx_{e}^{n})$	(8)

where: *A*, *B*, *C_BET_*, *k_GAB_*, *k*, *n*, *n′* are constants of the equations; *X_e_* is the equilibrium humidity (g water/g dry basis); *X_m_* is the humidity of the molecular monolayer (g water/g dry mass); *R* is the universal gas constant; and, *T* is the temperature (K).

**Table 3:** Model parameters for adsorption isotherms.

Model		Parameters		R^2^	SEE	MAE (%)	Residual Distribution
Temperature dependent							
GAB	18 °C	*X_m_*	0.076	0.967	0.013	5.149	Random
		*C_GAB_*	1,502,959				
		*K*	0.755				
	25 °C	*X_m_*	0.068	0.973	0.014	8.795	Random
		*C_GAB_*	4,501,090				
		*K*	0.825				
	30 °C	*X_m_*	0.064	0.984	0.011	8.508	Random
		*C_GAB_*	1,812,258				
		*K*	0.842				
BET	18 °C	*X_m_*	0.028	0.301	0.056	33.845	Trending
		*C_BET_*	−19.315				
	25 °C	*X_m_*	0.030	0.604	0.049	26.359	Trending
		*C_BET_*	−20.218				
	30 °C	*X_m_*	0.029	0.594	0.051	27.66	Trending
		*C_BET_*	−21.015				
Oswin	18 °C	*A*	0.132	0.959	0.014	6.657	Random
		*B*	0.264				
	25 °C	*A*	0.127	0.957	0.016	9.171	Slightly random
		*B*	0.323				
	30 °C	*A*	0.121	0.966	0.015	9.870	Slightly random
		*B*	0.345				
Modified Henderson	18 °C	*k*	0.336	0.912	0.020	10.794	Trending
		*n*	2.518				
	25 °C	*k*	0.130	0.903	0.024	12.346	Trending
		*n*	2.005				
	30 °C	*k*	0.095	0.927	0.022	12.53	Trending
		*n*	1.809				
Chun-Pfost	18 °C	*A*	−24.266	0.948	0.015	7.443	Random
		*B*	19.759				
	25 °C	*A*	−16.300	0.929	0.021	12.428	Trending
		*B*	16.826				
	30 °C	*A*	−13.974	0.940	0.020	13.612	Trending
		*B*	16.113				
Temperature independent							
Halsey	18 °C	*A*	0.002	0.974	0.011	5.902	Random
		*B*	2.867				
	25 °C	*A*	0.004	0.976	0.012	8.628	Random
		*B*	2.387				
	30 °C	*A*	0.005	0.975	0.013	10.412	Random
		*B*	2.276				
Henderson	18 °C	*k*	97.702	0.912	0.020	10.794	Trending
		*n*	2.518				
	25 °C	*k*	38.724	0.903	0.024	12.346	Trending
		*n*	2.005				
	30 °C	*k*	28.762	0.927	0.022	12.529	Trending
		*n*	1.809				

**Table 4:** Activation energy of the GAB isotherm parameters.

Parameters	18 °C	25 °C	30 °C	*E_a_* (kJ/mol)
*X_m_*	0.0764	0.0677	0.0640	−10.947
*C_GAB_*	1,502,958.98	4,501,089.95	1,812,257.53	18.843
*k*	0.7552	0.8252	0.8418	6.820

## Data Availability

The data presented in this study are available in this same article.
